# Variations in periplasmic loop interactions determine the pH-dependent activity of the hexameric urea transporter UreI from *Helicobacter pylori:* a molecular dynamics study

**DOI:** 10.1186/s12900-015-0038-0

**Published:** 2015-06-26

**Authors:** Javier Cáceres-Delpiano, Jaime Teneb, Rodrigo Mansilla, Apolinaria García, Alexis Salas-Burgos

**Affiliations:** Department of Pharmacology, School of Sciences, University of Concepción, Concepción, Chile; Department of Microbiology, School of Sciences, University of Concepción, Concepción, Chile

**Keywords:** *Helicobacter pylori*, UreI, pH, Molecular dynamics, Periplasmic loop

## Abstract

**Background:**

*Helicobacter pylori* is an important factor in the development of diseases such as ulcer and gastric cancer. This bacterium uses a periplasmic transporter, UreI, to deliver urea to the intracelullar space, where later it is transformed into ammonia by the cytoplasmic enzyme urease to survive the acidic condition of the human stomach. The UreI transporter presents a pH-dependent activity, where this pH-dependence remains unknown at a structural level. Althought the existance of several protonable residues in the periplasmic loops are related to the pH-dependent activity, we find interesting to have a clear view of the conformational changes involved in this phenomena through a molecular dynamic study.

**Results:**

Molecular dynamic simulations of the UreI transporter at three different pH conditions were performed, revealing two main pH-dependent conformations, which we present as the open and close states. We find that salt bridges between the periplasmic loops are crucial interactions that stabilize these conformations. Besides, a cooperative behaviour exists between the six subunits of the system that is necessary to fulfill the activity of this transporter.

**Conclusions:**

We found different pH-dependent conformations of the urea transporter UreI from *Helicobacter pylori*, which are related to salt-bridge interactions in the periplasmic regions. The behaviour of every channel in the system is not independent, given the existance of a cooperative behaviour through the formation of salt-bridges between the subunits of the hexameric system. We believe that our results will be related to the generation of new eradication therapies using this transporter as an attractive target, denoting that the knowledge of the possible pH-dependent conformations adopted for this transporter are important for the development of rational drug design approximations.

**Electronic supplementary material:**

The online version of this article (doi:10.1186/s12900-015-0038-0) contains supplementary material, which is available to authorized users.

## Background

*Helicobacter pylori* is a gram negative, neutrophil type and spiral shape bacterium. It develops in a microaerophilic environment, usually found in the stomach, and with its 4–6 flagella that allows it to traverse the gastric mucus, it can colonize the surface of gastric epithelial cells [1]. This pathogen is well adapted to its inhospitable niche, where one can see that after the first infection, chronic infection is developed, which can last several decades. Selecting this environment without competition and the ability to generate this chronic infection, makes *H. pylori* one of the most successful human pathogen. More than 50% of the world population has *H. pylori* in their stomach, being the largest etiological agent of gastric diseases such as chronic gastritis, peptic ulcer disease and MALT lymphoma. Furthermore, it has an important participation in gastric carcinogenesis (gastric atrophy, intestinal metaplasia, dysplasia and gastric adenocarcinoma) [2].

The successful colonization of the human stomach by *H. pylori* is possible by a combination of factors, such as virulence factor like exotoxin VacA [3] and the pathogenicity island cag PAI, encoding a type IV secretory system and the effector protein CagA [4]. Even with the presence of these virulence genes, one of the most important processes in the development and survival of *H. pylori* is the neutralization of acidic pH in the stomach. To colonize the gastro-intestinal tract, this bacterium has to survive the acidic lumen of this niche. This pathogen spends a lot of genes to the biosynthesis and maturation of a protein called urease, a Ni^2+^ dependent enzyme that hydrolyses urea to ammonia and carbon dioxide. The biosynthesis of urease is governed by a cluster of seven genes, including genes coding for the UreA and UreB subunits of this enzyme, and genes encoding for accessory proteins UreE, UreF, UreG and UreH, related to the capture of Ni^2+^ and its insertion in the active site of the apo-enzyme [5].

A previous step to the metabolism of urea by *Helicobacter pylori*, is the transportation of this metabolite to the intracellular space. In the 7 genes cluster, it has been identified the presence of a gene called *ureI*, who is not involved in the biosynthesis of urease. This gene encodes for a transporter called UreI, a proton dependent channel that transports urea from the periplasmic to the intracellular space. It has been demonstrated that strains of *H. pylori* deficient of this transporter are unable to colonize mice, in addition to a requirement of this transporter for resistance to acidic pH *in vitro* [6]. This positions UreI as a potential target for eradication therapies. This transporter is an inner membrane protein with molecular weight of 21.7 kDa and has no signal peptide. The transporter is closed at pH ~6.0 and fully open at pH 5.0 or less, allowing rapid transport of urea within the bacteria. This regulation permits the pH neutralization by intracellular urease and disables the over alkalization. The structure consists of 6 subunits, which together form a hexameric ring with a diameter of 95 Å and 45 Å height. Every one of the six subunits correspond to a single functional channel, where has been thought that each one functions independently to the other [7].

The *ureI* genes are found in gastric and oral bacteria, like *Helicobacter pylori* and *Streptococcus salivarius* [8], and more recently in the enteric bacteria *Helicobacter hepaticus*, encoded for a family of inner membrane urea transporters. This family has homology with six transmembrane segments amides transporters in bacteria, but not with the urea transporter family of ten segments present on multiple species of bacteria and mammals [9]. In the UreI transporters family, the six transmembrane segments are approximately 85% of sequence identity, where the cytoplasmic domains are also conserved. While the periplasmic domains have 24 and 47 amino acids not conserved with *H. pylori*, from *S. salivarius* and *H. hepaticus*, respectively. Furthermore in *H. pylori* the urea transporter mediated for UreI is activated at low pH, whereas in the *S. salivarius* is independent of pH [10].

Apparently the protonation/deprotonation of specific amino acids are the basis for turn on/off the substrate transporter in this channel. Furthermore, it has been found that cytoplasmic pH is kept constant through a wide range of acidity [11] and the only protonable residue retained on the membrane of the insensitive UreI protein of *Streptococcus salivarius* is Glu177, concluding that the residues that respond to pH in *H. pylori* UreI should be in the periplasmic region [12].

McNulty et al. [13] revealed a comprehensive mechanism of the urea transport through the UreI channel at atomic resolution. They found two constriction sites in the central channel of this protein, which are related to the selectivity for urea (residue Trp153) and the pathway orientation of the substrate along the channel (residue Tyr88). Unfortunately there is no information about possible conformations of this transporter and its activity states.

In this work, we found different conformation of the periplasmic loops of the urea channel UreI from Helicobacter pylori through the use of molecular dynamics simulations at distinct pH conditions. We find evidence of certain group of residues involved in the conformation adopted by these regions at every condition. As believed, the existence of protonable residues in the periplasmic regions, such as histidine or aspartic/glutamic acid, play a fundamental role in the structure adopted, mainly through the formation of salt bridges. Our analysis also rejects the hypothesis of an independent behaviour of every subunit of the hexameric system, describing conserved interactions between each channel that are related to the stabilisation of the observed conformation. Furthermore, we conclude that these results will affect directly the future of structure based rational drug design approximation, using this transporter as an attractive target and the information of the different pH-dependent conformations.

## Methods

### Loop modelling

The model of a the PL1 loop of UreI was modelled using the one protomer of the trimeric hpUreI crystal structure (3ux4). The missing residues between 59 to 73 where modelled based on the hpUreI sequence (P56874) using the program Modeller v9.14. After this we performed a loop refining protocol, also with Modeller v9.14 [14].

### System building

The hexameric channel arrangement of hpUreI was built using the BuildMolecules module of the Python package ProDy [15]. Crystallization waters were added to the hexameric system using the DOWSER software [16], then embed the protein in a 160 Å × 160 Å palmitoyloleoyl-phosphocholine membrane (POPC), wrapped up in a TIP3 water box, and add Na + and Cl- ions to a concentration of 150 mM. This was repeated to all systems, with a total of ~200.000 atom for each. Protonation states of ionizable residues were determined using the propka server (http://nbcr-222.ucsd.edu/pdb2pqr_2.0.0/) (see Additional file 1). According to the desired pH of the model (2.0, 6.0 and 7.4), the residues were patched using the psfgen package of the VMD program [17] at the moment of the system construction.

### Molecular dynamics simulations

Molecular dynamics simulations were performed using NAMD 2.9 [18], with the CHARMM36 force field parameters. Firstly, the system was subject to a first minimization where only the movement of the acyl group of POPC bilayer was allowed, to induce the appropriate disorder. A second minimization was performed, where the movement of all environmental regions (lipid, water and ions) and the side chains of the channel were allowed. The movement of water molecules was restricted in direction to the membrane region using the NAMD Tcl forces module. The last minimization consisted in the movement of the entire system, with no restrictions. Each minimization was followed for 5 ns of equilibration, with a total of 15 ns. Finally, the production runs consisted in 20 ns for every model of the hpUreI system. A 12 Å cutoff distance for Van der Waals interactions using periodic boundary conditions was applied. Equations of motion were integrated at a 2 fs time step period. The temperature was maintained at 300 K with Langevin dynamics and the pressure was set to 1 atm. Long- range electrostatic interactions were computed by employing the smooth Particle Mesh Ewald (PME) method with a number of grids in each dimension of 162×162×93. All simulations were analyzed using the ProDy package of Python and the VMD software.

### Free energy maps

In order to elucidate local minima of the different conformations adopted for the hpUreI system, relative free energy maps were built, based on the methodology described in Minkara et.al. [19]. Briefly, the maps were constructed based on the probability to find a coexistence of the distance between pair of residues forming salt bridges in any of the 2000 frames computed during the simulations. This is, the times that a distance is repeated during the simulation versus the total frames of the simulation. We can extract values of relative free energy from these probabilities based on the Eq. 1:

1 $$ \varDelta \mathrm{G}\_\mathrm{r}\mathrm{e}\mathrm{l}=\hbox{-} \mathrm{RTLn}\left(\mathrm{Pi}\right) $$

Where R is the ideal gas constant, T the temperature and Pi the probability of coexistence of distances between two pairs of residues. The calculated distances were based on the α-carbon of the specified residues.

## Results

### Trajectory analysis of hpUreI system

**Fig. 1:**
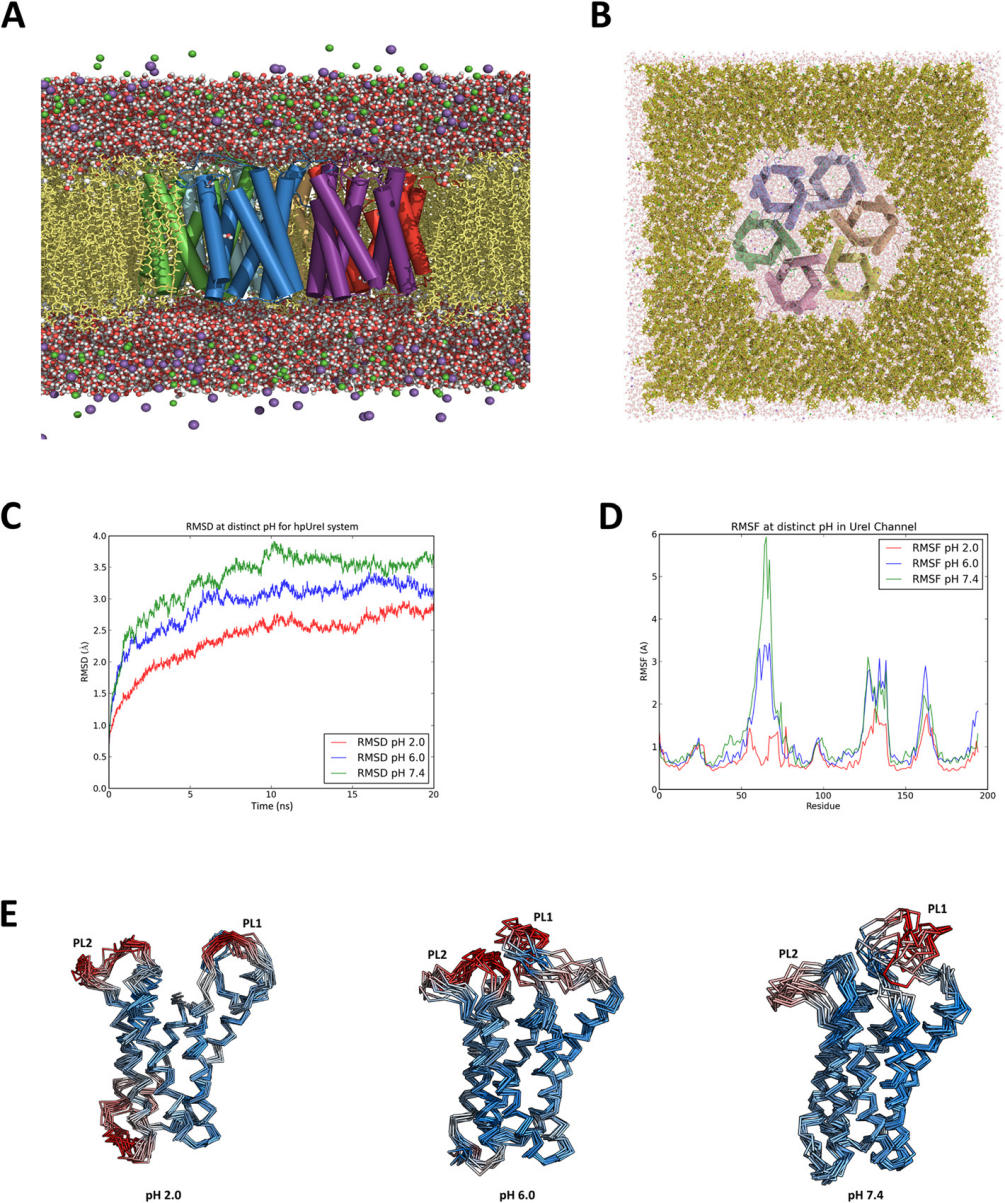
Trajectory analysis of the hpUreI system. **a** Front view of the hpUreI system embedded in a popc lipid bilayer, solvated in presence of 150 mM of NaCl. **b** Up view of the hpUreI system. **c** RMSD (Å) values for the full hpUreI system. RMSD values were calculated for each frame of the simulations at pH 2.0 (*red*), pH 6.0 (*blue*) and pH 7.4 (*green*). **d** RMSF values for a single UreI channel extracted from the entire system at pH 2.0 (*red*), pH 6.0 (*blue*) and pH 7.4 (*green*), showing the response of the two peaks against changes on pH condition of the system. **e** Structural alignment of a single subunit of hpUreI complex at different times of simulation, colored by flexibility. The figures were rendered with the visualization software PyMol

We built an hpUreI system embedded in membrane, solvated and ionized (Fig. 1a,b), to evaluate the behaviour of the channel through molecular dynamic studies. As shown in Fig. 1c, RMSD was calculated for the three full systems at every pH condition. As can be seen, the RMSD values increase in a dependent manner with the simulated pH, and the expected plateau is formed around the last 10 ns of the simulation. At pH of 2.0, the maximum value found was 2.94 Å and the maximum values for the other two conditions were 3.42 Å and 3.92 Å, at of 6.0 and 7.4, respectively. As displayed, only a modest increase can be seen at ~15 ns for the condition of pH 2.0 and pH 6.0, compared with bigger fluctuation observer at pH 7.4.

RMSF calculations were performed to evaluate fluctuations of the system at specific regions and to see if these are related to pH-dependent structural changes. All the subunits of the system present two major peaks, located in the regions given by residues ~50-80 (red dot in Additional file 2) and ~120-140 (blue dot in Additional file 1). A third peak was observed, corresponding to a cytoplasmic region that was not studied in this work. In a more detailed analysis, Fig. 1d shows the calculated RMSF for only one channel of the entire system as a representative manner, showing that these regions coincide with the location of the periplasmic loops PL1 and PL2. Furthermore, is shown that the behaviour of RMSF values are also pH dependent, seeing an increase from 1.5 Å for the system at pH 2.0 to values of approximately 3.0 Å and 6.0 Å for the system at pH 6.0 and 7.4, respectively. These values can be represented as a marked increase in the mobility of this loop as the pH of the system increases. With respect to the periplasmic loop PL2, no significant changes in all subunits are observed, with the exception of the chain E, which has a higher mobility compared to other subunits (see below). The results of fluctuation are clear in relation to the periplasmic loops PL1 and PL2, seeing a strict pH dependency on its behaviour. Fig. 1e shows structural alignments at different times of the molecular dynamic simulation for only one subunit of the complex. It can be seen an orderly motion for loop PL1 at pH 2.0 and a clear movement of the PL1 region towards the PL2 at pH 6.0, while for the PL1 structure at pH 7.4 the behaviour is messy and follows no pattern.

### Distance between periplasmic loops

**Fig. 2:**
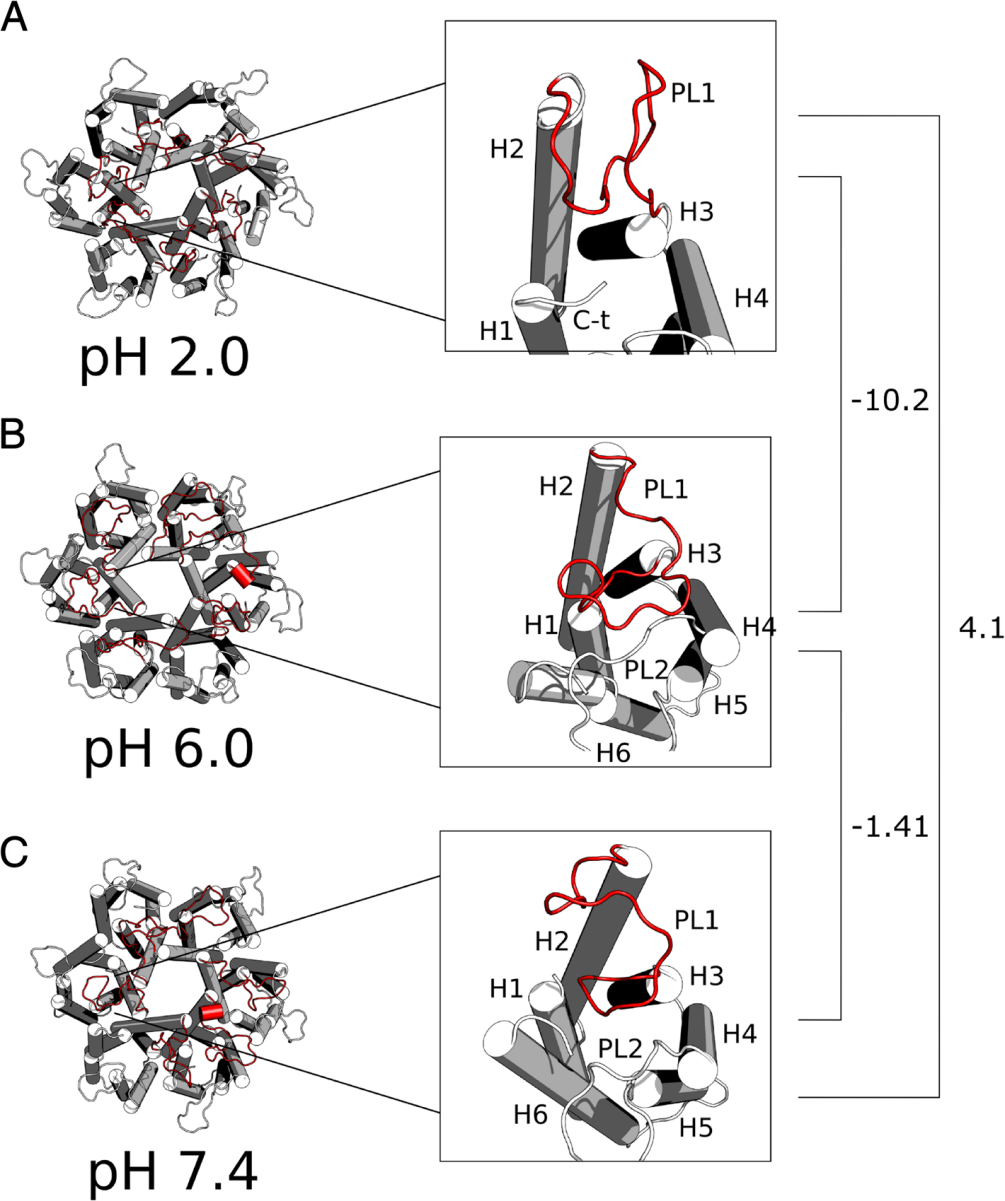
The different orientations of the periplasmic loop. **a** UreI at pH 2.0 and a zoom to the trapped conformation, facing the adjacent chain against clockwise. **b** Conformation of UreI at pH 6.0 and enlargement of the conformation observed related to the movement of PL1 loop toward PL2 (**c**) Orientation of PL1 loop at pH 7.4. It is observed that the PL1 loop loses coherence within the system, showing no pattern in their orientation. At the right, the covariance values are annoted for each pair of conditions

Different changes can be seen in the structure of the hpUreI system that is mainly involved in the movement of the periplasmic loops, especially in the periplasmic loop PL1 (Fig. 2). The PL1 loop at pH 2.0 is in a "trapped" like conformation towards the interior of the complex, and adjacent to the next subunit in a counter clockwise direction (Fig. 2a). This, in comparison to the structure at pH 6.0, shows a release of this trapped conformation of PL1, now oriented towards the PL2 loop of the same subunit (Fig. 2b). In contrast, the structure at pH 7.4 shows no coherence between each of the subunits, where further analyses are required (Fig. 2c). In order to evidence how these variables change in concordance with one each other, we decide to make covariance analysis between pair of conditions. The calculate values of covariance between pH2 and pH6 (covariance = −10.2) and the pH6 and pH7 pair (covariance = −1.41), presents a negative value, suggesting an opposite behaviour, which in our case is related to an open or a closed state of the channel given by the distance between periplasmic loops. It is interesting to see that the value of covariance between pH2 and pH7 presents a positive sign (covariance = 4.1) that might be interpreted as a similar behaviour in both conditions, suggesting that in both condition an open conformation exists.

**Fig. 3:**
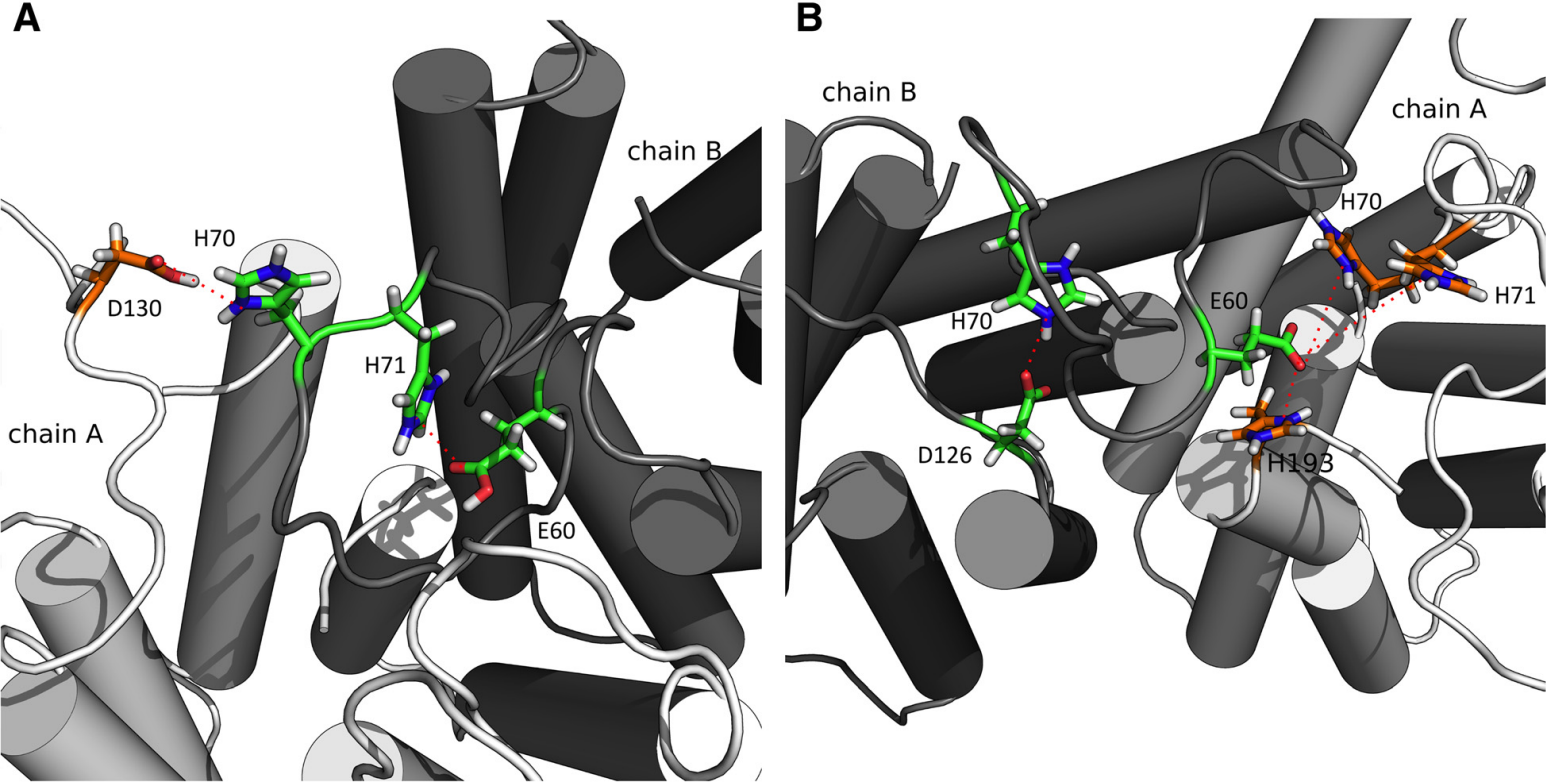
Schematic representation of salt bridges. **a** Possible interaction between intra-loop residues and inter-chain residues at pH 2.0. **b** Interactions between inter-loop residues and inter-chain residues at pH 6.0. Red dashed lines represent which residues are interacting. The figures were rendered with the visualization software PyMol

**Fig. 4:**
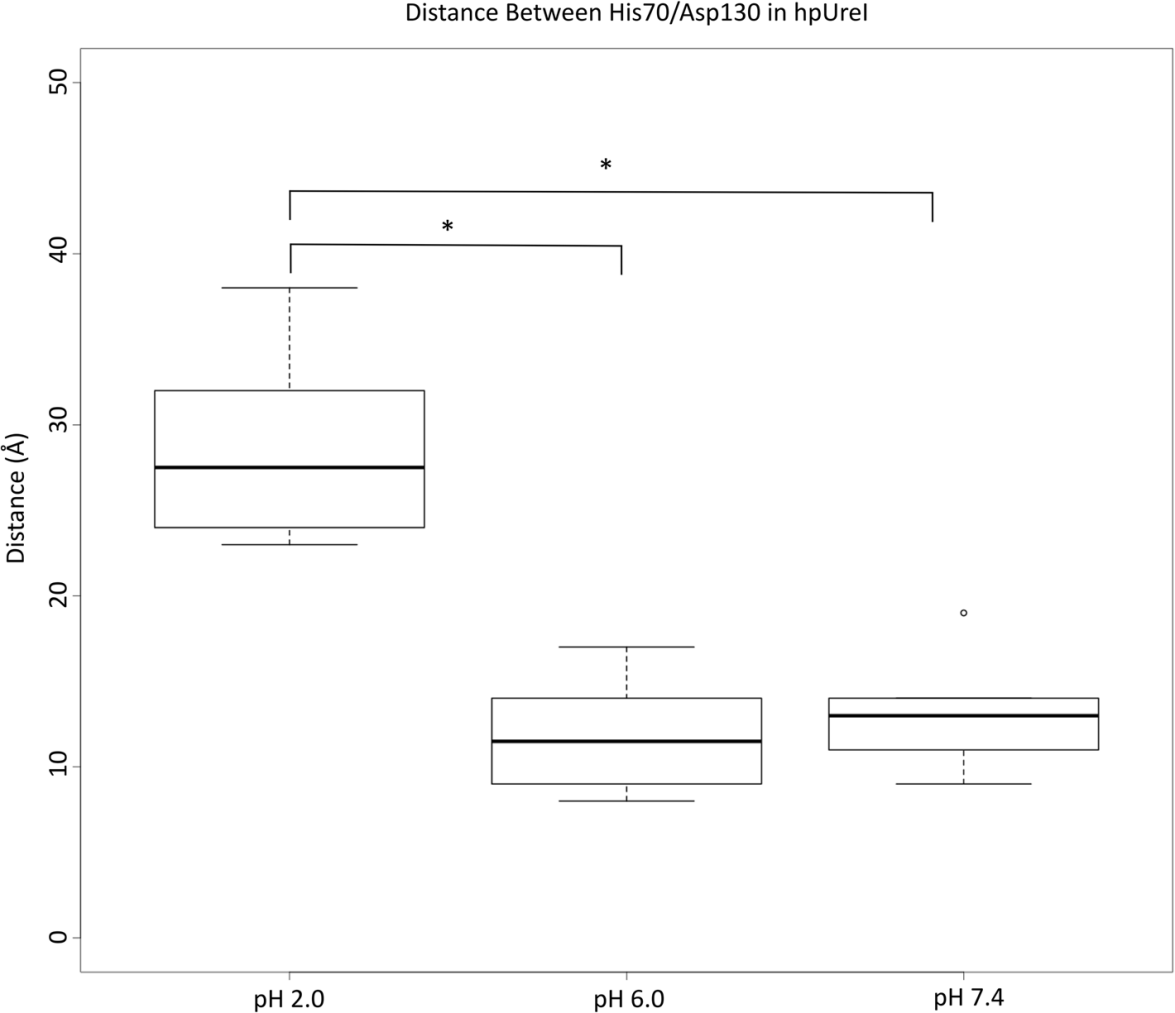
Distance variations between periplasmic loops. Average distances of the loops of each subunit of the full hpUreI system at each pH condition were calculated and box plotted. The values were represented as the distance between residues H70 and D130 from PL1 and PL2, respectively. As can be seen, de distance of periplasmic loop at pH 2.0 is greater than the distance observed at pH 6.0 and 7.4. Statistical significance were calculated using t-test, where the groups pH 2.0/pH 6.0 and pH 2.0/pH 7.4 show a p-value < 0.05 (p = 0.00027 and p = 0.00046, respectively)

Based on the hypothesis of a possible interaction between the periplasmic loops, measuring of the distance between the periplasmic loops for every subunit of the full system will give us a quantitative view (Fig. 4), compare to the qualitative view in Fig. 3. The chosen residues were His70 for PL1 and Asp30 for PL2. Average values for the distance on every subunit were taken and graphed as shown (Fig. 4). The results are consistent with the expected for all simulations showing at pH 2.0 the average distance is ~27 Å, which is ~15 Å greater than the system at pH 6.0. These differences are also statistically significant, showing us that this event is probably due to pH condition, rather than a simple chance. There is no statistical significance difference between the obtained values for pH 7.4 and pH 6.0 simulations. It has to be noted that the behaviour of the system at pH 7.4 is different from others, showing an extreme value for one of the subunits. We interpreted this as a greater flexibility in the loop regions for this condition and to a loss in coherence of the system response against pH changes.

### Salt bridges controlling loop motion

The periplasmic region of the hpUreI system contains numerous ionisable residues, where the pH may play a crucial role. One of the main interactions in protein stabilization, which are also sensitive to changes in proton concentration, are salt bridges. Distance between oxygen and nitrogen for protonable residues in every subunit of the hpUreI system during the simulations were analysed for all the three pH conditions, and only residues conserved in more than 75% of the simulations were annotated in Table 1. Focused primarily on PL1 and their "trapped" conformation at pH 2.0, four residues that could possibly be involved in the formation of salt bridges for all channels of the hexamer complex were found. The Asp60 and His71 atoms are in a distance < 4.0 Å within the same loop PL1 for all channels within the complex, with an exception for chain C, which can form a bridge between Asp60 and His54 residues. Furthermore, the His70 residue is capable of forming a salt bridge with Asp130 residue of the adjacent chain (Table 1). Besides the fact that these type of interactions between residues exist in all the channels of the complex, it is interesting to notice that the existence of the participation of inter-loop residues and also residues from adjacent subunit, will play a crucial role in the stabilisation of the conformations.

**Table 1:** Residues involved in salt-bridge interactions. Residues that are capable to form a salt bridge in the periplasmic regions of hpUreI system. This information are show for every chain in all the three pH simulated

	Chain A	Chain B	Chain C	Chain D	Chain E	Chain F
pH 2.0	E60 – H71	E60 – H71	E60 – H54	D126 – H131	E60 – H71	E60 – H71
	D129 – K132	D130 – H70_F	D126 – K132	D130 – H70_E	D130 – H70_C	D130 – H131
	D130 – H131	E138 – H131	D129 – H131	D140 – H123	D130 – H131	E138 – K132
	D130 – H70_B		D129 – K132		D140 – H131	
					D140 – H123	
pH 6.0	E60 – H193	E60 – H71_A	E60 – H71	E60 – H70_F	E60 – H71	E60 – H71
	E60 – H131_C	E60 – H193_A	E60 – H54_A	E60 – H54	E60 – H193_D	E63 – K132_B
	E60 – H70_C	E60 – H70_A	E63 – H193_E	E60 – H71	D64 – H70	E63 – H71
	D126 – H131_C	E63 – H70	D64 – H70	D64 – H71	E63 – H70_D	E63 – H193_B
	D126 – H193_C	D126 – H70	D129 – H71	E63 – H193_F	E63 – H131_D	D64 – H71
	D129 – K132	D129 – H70	D130 – H193	E63 – H70	D129 – K132	D126 – H123
	D130 – H193	D129 – H71	D130 – H131	E63 – H71	D130 – K132	D129 – H131
	D130 – H70	D130 – H193	D130 – H70	D126 – H123	D130 – H71	D129 – K132
	D130 – H131	D130 – K132		D126 – H71	D130 – H131	D129 – H70
	D130 – H71	D140 – H131		D130 – H131	D140 – H123	D130 – H131
	E138 – H123	D140 – H123		E138 – K132	D140 – H131	E138 – K132
	D140 – K132			D140 – H123		
pH 7.4	------	D130 – K132	E138 – K132	D130 – K132	E63 – K132	D130 – K132
					E138 – K132

In case of the system at pH 6.0, where most of the aspartic and glutamic residues are deprotonated, the analysis was focused on those inter-loop residues, in addition to analysing a possible inter-chain collaboration. It was found that the His70 and His71 from PL1 could form salt bridges with Asp126, Asp129 and Asp130 residue from PL2. Regarding the inter-chain interactions, it was found that Asp60, Asp63 and Asp126 residues could form salt bridges with the His70, His71, His131 and His193 residues from the adjacent chain (Table 1). This also gives us strong evidence to establish the hypothesis of full system collaboration to stabilize each conformation, rather than just being stabilized from an interaction between periplasmic loops.

In relation to simulation at pH 7.4, where the majority of residues are deprotonated, salt bridge interactions of any inter-loop or inter-chain residue were not found. Salt bridge interactions between residues Asp130 and Glu138 with Lys132 residue from PL2 loop were found, but no biological meaning in relation to the stabilisation of open/closed conformation was concluded. This could demonstrate the existence of a limit of the behaviour of this system strictly related to the pH conditions.

### United behaviour of the hpUreI system

**Fig. 5:**
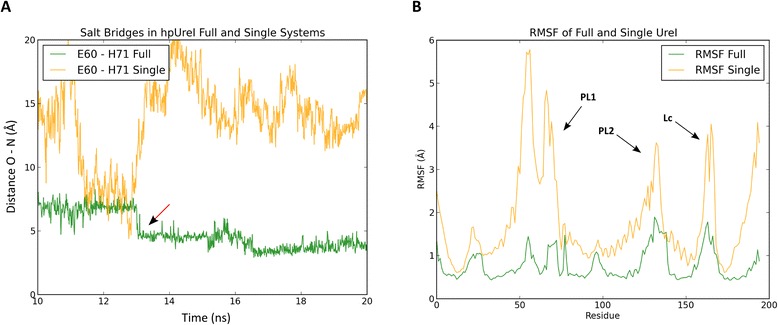
United behaviour in the hpUreI system stability. **a** Distance between the ε2 atom of the Asp60 residue and the δ1 or ε2 atom of His71 for the hexamer system (green) or a single monomer (orange). A variable behavior in the distances for the monomer are observed, compared to the hexameric system. The arrow shows when the distance is observed at ≤ 4.0 Å. **b** Comparison of the RMSF values for a monomer of the full hpUreI system and the results of the simulation of only one monomer

In order to study the importance of the hexameric arrangement of the hpUreI system, and to see if the observed coherence in the formation of inter-chain salt bridges is necessary to stabilize the “trapped” conformation, a molecular dynamics simulation of only one monomer of hpUreI system protonated at a pH 2.0 condition was performed. As previously mentioned, there are four important residues that potentially form salt bridges at pH 2.0, probably stabilizing the open conformation. This corresponds to the salt bridge between residues Asp60 and His71 in an intra-loop manner, and the salt bridge between residues Asp130 from one chain and His70 of the adjacent chain in a counter clockwise direction. Monomer simulation clearly eliminate the salt bridge between Asp130/His70, giving us a chance to evaluate if the absence of this salt bridge destabilizes Asp60 and His71 interaction, interpreting this in terms of flexibility. As shown in Fig. 5, a change in the distance between the Asp60 and His71 is clear. The simulation of a single monomer shows varied changes in the distance between these two residues, and never reaches a smaller or equal distance to 4.0 Å required for the classification of and interaction as a salt bridge. Furthermore, we show that the non-formation of this salt bridge increases the flexibility for both PL1 and PL2 loop. This allows us to widely conclude that the hexameric conformation of the system, as well as the formation of salt bridges, such as Asp60/His70, are key steps in the process of stabilization of the open and/or closed conformations.

### Free energy maps of the hpUreI system

**Fig. 6:**
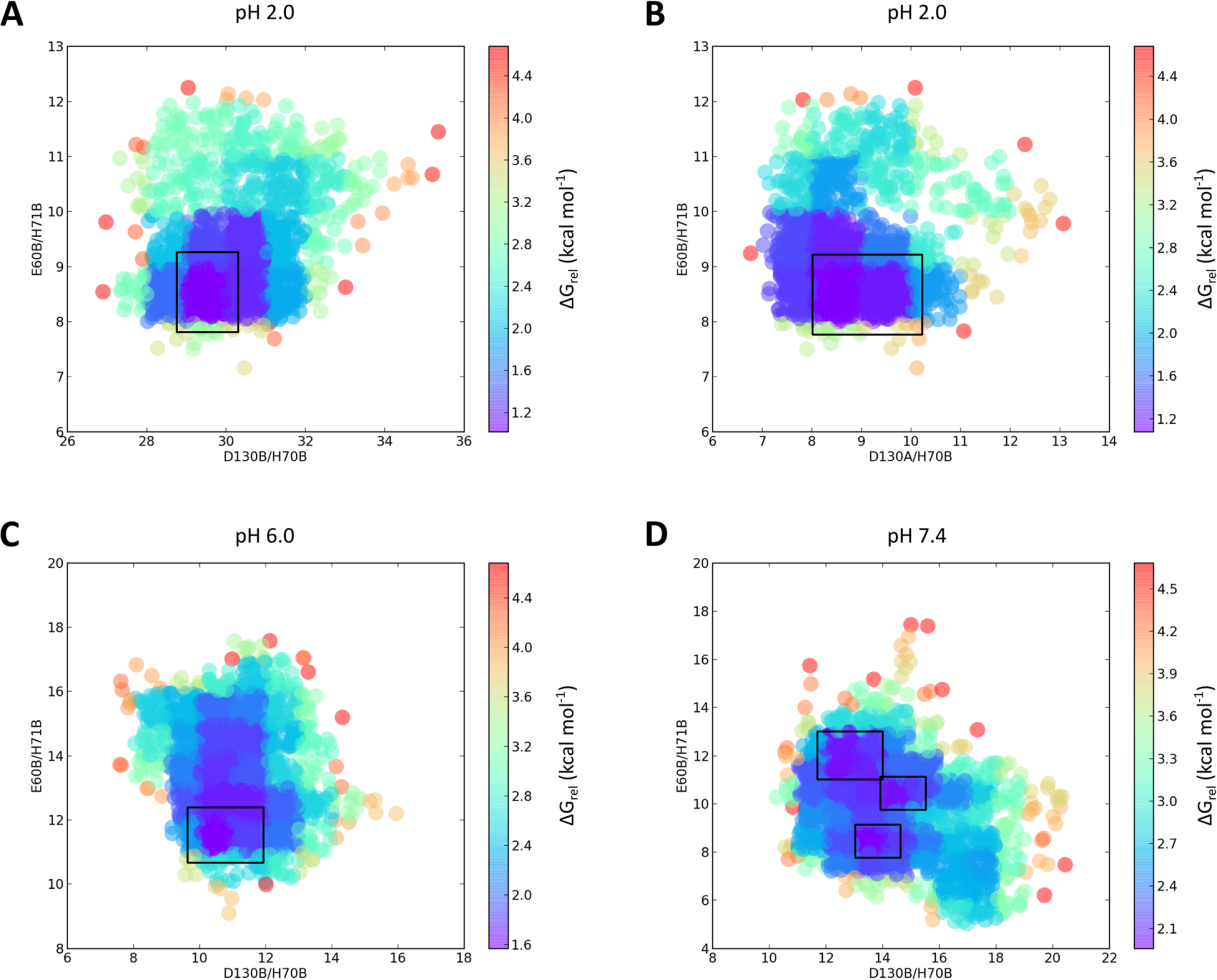
Relative free energy maps for the hpUreI system at distinct pH conditions. **a** Energy map at pH 2.0 based on the distance between inter-loop and PL1 intra-loop residues. **b** Energy map at pH 2.0 based on the distance between inter-loop and inter-chain residues. **c** Energy map at pH 6.0 based on the distance between inter-loop and PL1 intra-loop residues. **d** Energy map at pH 7.4 based on the distance between inter-loop and PL1 intra-loop residues. Local minima are represented in black boxes for every energy map

In order to elucidate local minima in every pH condition, we constructed free energy maps based on the probabilities to find pair of residues at a specific distance through all the frames of the simulations. At pH 2.0 we use the pair of residues Glu60_chainB_/His71_chainB_ vs. Asp130_chainB_/His70_chainB_ or Asp130_chainA_/His70_chainB_. As can be seen, clear local minima exists for both maps (Fig. 6a,b), where the distance of α-carbon between residues of the periplasmic loops is ~29 Å, and for the intra-loop residues Glu60 and His71 is ~8 Å. At pH 6.0 the produced map was between the same groups of residues used at pH 2.0 (Fig. 6c). In comparison to the distance observed at pH 2.0, the distances between the periplasmic loop decreases to ~10 Å, and for the intra-loop residues the distance increase to ~11 Å. The most interesting finding can be seen in the free energy map at pH 7.4. Unlike the other two conditions which has a clear single local minima, the system at pH 7.4 exhibits three local minima represented in Fig. 6d. This behaviour can be refered to an increase in flexibility of the periplasmic regions, which pass through a variety of conformation, where the upper local minima in Fig. 6d represents the closest condition of a close conformation, similar to the distance observed at pH 6.0. The other two local minima show an increase of ~2-4 Å in the distance of the periplasmic loops, and a decrease of the distance of the inter-loop residues to distance close to the observed at pH 2.0, which can be referred to an open conformation. This variation in the conformation observed at pH 2.0 and 6.0, in comparison to pH 7.4, shows a limit in the functionality of the channel in relation to pH condition, assuming that a clear and constant conformation is important.

## Discussion

**Fig. 7:**
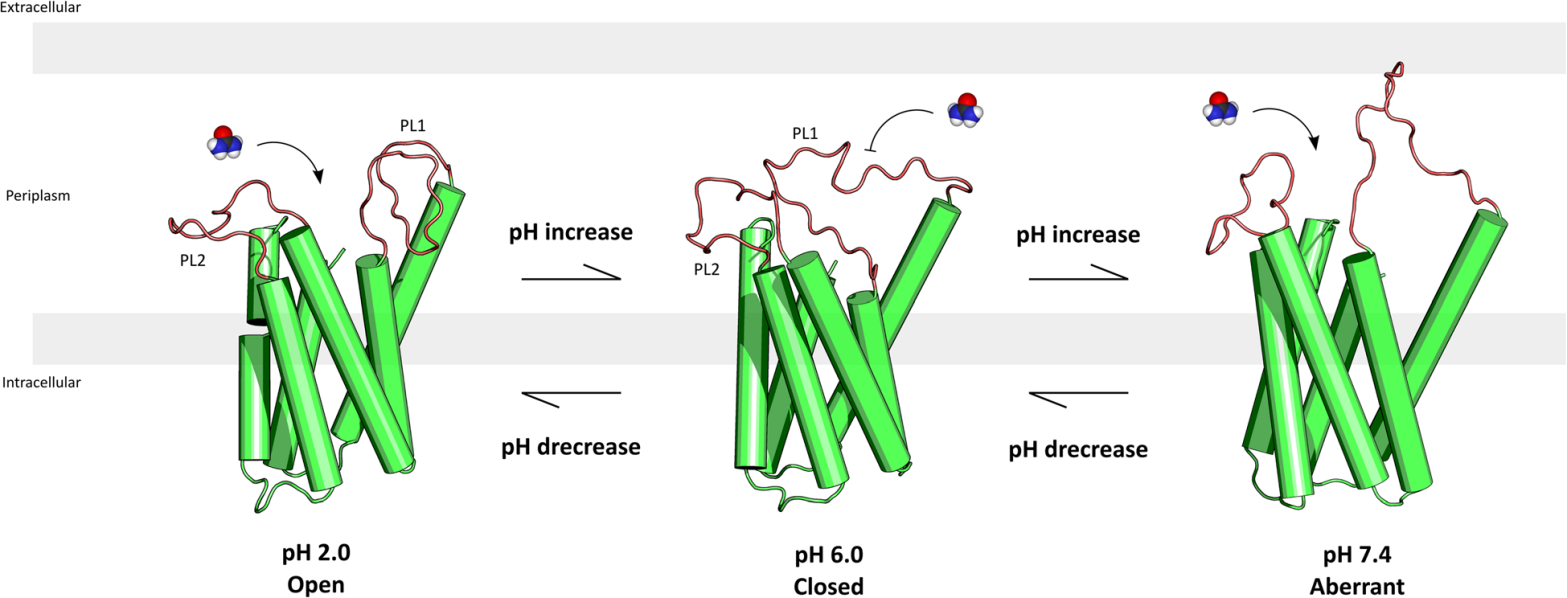
Schematic representation of the pH-dependent conformations. Cartoon representation of one protomer of the hexameric hpUreI complex at each pH condition. Periplasmatic loops are colored in red to better discriminate conformational changes, and how the structural changes might affect urea transport

We have reported molecular dynamic simulation of the hpUreI channel at distinct pH condition, in order to find differences on the structure that explain the pH-dependent function of this transporter. According to our results related to the behaviour of systems at different pH, dramatic changes are observed in the periplasmic loops. These loops have great flexibility, which increases proportionally to pH. This increased flexibility is quantified by the increase in RMSF values, which increase from 1.5 Å at pH 2.0 to two and four fold at pH 6.0 and pH 7.4, respectively. Moreover, the orientation of the periplasmic loops significantly changes when the pH of the system is altered. At pH 2.0 the periplasmic loop PL1 is oriented towards the adjacent chain trapped and with low flexibility. Upon increasing the pH, this loop is released from its trapped conformation, now moving towards the PL2 region. If the pH increases further, in our case at pH 7.4, the PL1 region becomes more flexible with an errant behaviour and inconsistent motion (Fig. 7). Regardless of the statistical significance in the distance of the periplasmic loops, the distances at pH 7.4 have a higher average distance, compared to the condition at pH 6.0, and present a subunit with an extreme value for the distance that escapes the "normal" behaviour. With this, it is clear that the flexibility of the PL1 loop is pH dependent, giving way to study what kind of molecular interaction are key to this behaviour.

The analysis of salt bridges shows a consistent relationship with previous experimental results in literature. According to the study of Weeks and Sachs (2001), through the use of site-direct mutagenesis and the construction of chimeras between hpUreI and the pH independent UreI of *Streptococcus salivarius*, possible conclusions of the individual function of each of the periplasmic loops in relation to the opening and closing of the channel were established. They show that mutations in residues His70 and His71 generate an open conformation of the channel at neutral pH, in addition to reducing transport at acidic pH. First, this latter feature can be well explained mainly by the formation of salt bridges between residues Asp60/His71 and Asp130/His70 adyacent. It is believed that the formation of these salt bridges is critical to maintaining the trapped conformation of PL1 toward the adjacent chain. The trapped PL1 is the key to open channel conformation, enabling the transport of urea. With this, if the His70 and His71 are mutated (e.g. glutamine, as is done in the study of Week and Sachs, 2001), these interactions are lost, releasing the loop from its open conformation and urea transport difficult at acidic pH. Second, the generation of an open conformation at neutral pH can be explained through the formation of salt bridges between the His70 and His71 residues with Asp126, Asp129 and/or Asp130 residue from PL2. These interactions are crucial in maintaining periplasmic loops together, adopting the closed conformation. If these interactions do not exist, a high flexibility in the periplasmic loops and a fully open channel will form.

Another important conclusion in the study of Weeks and Sachs is the establishment of the PL1 loop as an important region in the maintenance of the closed conformation, a conclusion given by the construction of chimeras with the UreI of *Streptococcus salivairus*. Based on the results of this work, we found that the PL1 region is key to maintain both, the open and closed conformation, given by the formation of salt bridges intra-loop and inter-chain, and the formation of salt bridges with inter-loop PL2.

In the study of Weeks and Sachs [10], various mutations at His123, His131, Asp129, Glu138 and Asp140 residues set these as important for the transport of urea. Based on the results, we cannot directly determine the relationship of these residues to the transport of urea, but we can infer that these are involved in maintenance of the conformation of the open channel at acidic pH, as exemplified by the formation of a salt bridge between residues Asp130 and His70 adjacent. Mutations in His193 do not affect the open conformation to acid pH, such as His193Lys mutation. Based on the results of this work, the His193 residue is presumably forming a salt bridge with Asp60 or Asp63 of the same chain, or the adjacent chain, in addition to being an option in some cases to create a salt bridge with Asp126 of the adjacent chain or Asp130 of the same chain. Seeing that this system works in coherence between each of the chains of the system to keep each of the conformations, it is not surprising that the His193 is also part of this group of cooperative residues. An interesting case is the His193R mutation, which drastically reduces the transport of urea at acidic pH. The positive charge is maintained, but apparently the arginine residue interacts with some residue of the PL1 loop, based on the fact that interaction between the periplasmic loops is key in the regulation of the open/closed conformation. Still, it is too soon to draw conclusions about this behaviour since the results of this work do not help to elucidate this. Finally, chimeras of the PL2 loop of *H. pylori* with *Streptococcus salivarius* PL2 are inactive. This is clear, because the loop PL2 is responsible for stabilizing the loop PL1 through a cooperative action.

One last achievement was the necessary importance of united behaviour within hpUreI system, which is responsible for stabilizing each of the conformations. This work shows that the open conformation is stabilized strictly forming intra-loop and inter-chain salt bridge. The residues Asp60 and His71 are responsible for forming a salt bridge within the loop PL1, while the residue His70 of the same loop is able to interact with the Asp130 residue of loop PL2 of the adjacent chain. The formation of these interactions greatly stabilizes the system, significantly reducing the flexibility of the system compared to a system where only one monomer of this complex is simulated. McNulty et.al [13] shows that the PL1 loop move into the vestibule at the moment they performed the simulation of only one protomer. These changes may be explained by the pH conditions on which the simulation were performed (pH 6.1), and possibly by the presence of urea. At this pH, based on our simulations, one would expect the closure of the channel based on the protonation states of the periplasmic residues. Our simulation at pH 6.0 of the hexameric complex shows the formation of salt bridges between both, the periplasmic loops within one subunit and the periplasmic loops from adjacent subunits. Our simulation of one protomer was performed at pH 2.0, and show a relationship of the high RMSF values obtained for the PL1 loop and the statements from McNulty et.al of a possible cooperative behaviour given by the fact that the PL1 loop a single protomer is unstable and block the channel at pH less than 5.5.

## Conclusions

In this study we achieved to elucidate different conformations of the pH-dependent urea transporter UreI *from Helicobacter pylori* through the use of molecular dynamic simulations at different pH conditions, giving a structural view of previous experimental studies. The main observed changes were located in the periplasmic regions, were two periplasmic loops with high flexibility experienced different orientations in a pH-dependent manner, discriminating between an open and closed conformation. The main interactions that ruled this conformational changes were salt-bridges, that through a process of protonation/deprotonation of especific residues in the periplasmic loop will permit the creation and rupture of interacting residues, such as histidine, aspactic and glutamic acid. This type of interaction were found in the inter-loop region, intra-loop region and also in the inter-chain region, showing a united behaviour of the hexameric arrengement to stabilise the different conformation related to the activity of this transporter. We showed that the stabilisation of one transporter given the existance of its neighbourght is critical, and the absence of this cooperativity will lead to an increased flexibility of the transporter and a malfunction. The open conformation at acidic pH opens a door to the development of new rational drug design approximations, using this structure as base in the finding of possible molecules that could block the transport of urea. Also, the knowledge of a possible malfunction of the complex given the non-existance of a hexameric arrengement will be useful for the search of small-molecular that could block the interface between each subunit. These approaches are strong, and could be the basis for future rational drug design against this transport, inhibiting an important pathway in the survival of Helicobacter pylori in the human stomach.

## Additional files

Additional file 1:
**Modified residues in the periplasmic region.** Protonation states of protonable residues for each simulated system the hpUreI system. Additional file 2:
**RMSF values for entire hpUreI system.** Dashed lines splits the region between each subunit of the hexamer. Red and blue dot represents the PL1 and the PL2 region, respectively.

## Abbreviations

RMSD: Root mean square deviation
RMSF: Root mean square fluctuation
hpUreI: Hexameric UreI system from *Helicobacter pylori*
PL1: Periplasmatic loop 1
PL2: Periplasmatic loop 2
