# Supplementary figures and images for: Variations in periplasmic loop interactions determine the pH-dependent activity of the hexameric urea transporter UreI from Helicobacter pylori: a molecular dynamics study
Source: BMC Struct Biol. 2015 Jun 26;15:11. doi: 10.1186/s12900-015-0038-0 (PMC4482100; doi:10.1186/s12900-015-0038-0)

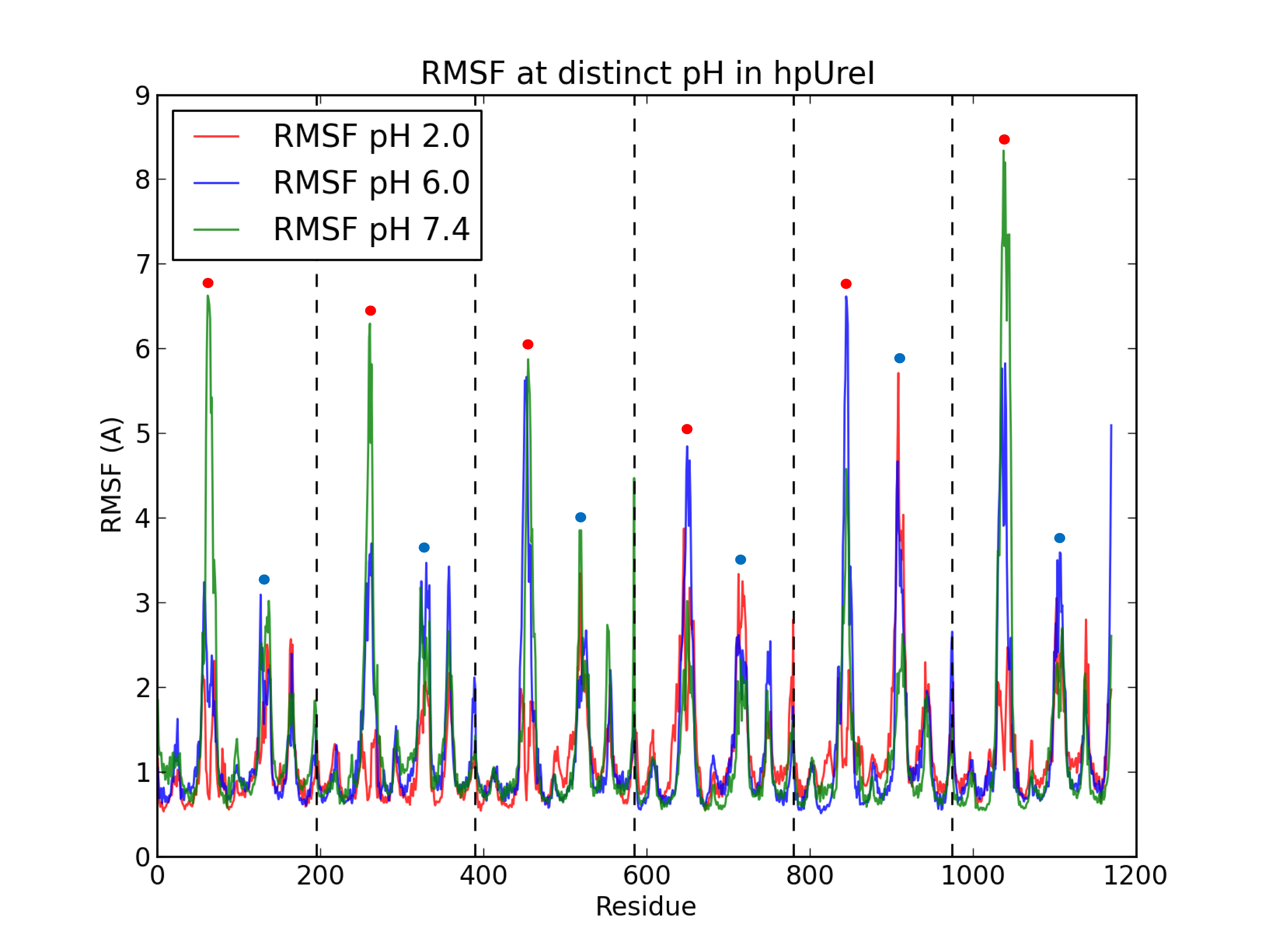

Supplement: Additional file 2: — RMSF values for entire hpUreI system. Dashed lines splits the region between each subunit of the hexamer. Red and blue dot represents the PL1 and the PL2 region, respectively. [file 12900_2015_38_MOESM2_ESM.png]
